# Integrated Electrocardiography-Heart Rate Variability (ECG-HRV) Analysis in Neurologically Symptomatic Individuals Without Cardiac Symptoms: A Data-Driven Retrospective Observational Study of Electrocardiographic-Autonomic Associations

**DOI:** 10.7759/cureus.112313

**Published:** 2026-07-09

**Authors:** Manju Lata, Manoharan Renugasundari, Gagan K Banodhe, Md Zabihullah, Tribhuwan Kumar

**Affiliations:** 1 Physiology, All India Institute of Medical Sciences, Patna, Patna, IND; 2 Physiology, Sri Manakula Vinayagar Medical College and Hospital, Puducherry, IND

**Keywords:** cardiac asymptomatic, electrocardiography (ecg), heart rate variability (hrv), neurocardiac inteaction, neurological signs and symptoms

## Abstract

Background and objective

Neurocardiac interaction is bidirectional, with neurological disorders influencing cardiac electrophysiology through the autonomic nervous system (ANS) pathways. Patients may present with central nervous system (CNS) manifestations, autonomic symptoms, or both. Despite the absence of overt cardiac symptoms, subtle autonomic disturbances may precede clinically detectable cardiovascular dysfunction. This study aimed to evaluate cardiac electrophysiology using heart rate variability (HRV) and electrocardiography (ECG) data from neurologically symptomatic but cardiac-asymptomatic individuals; to assess correlations between ECG parameters and HRV indices; and to determine the utility of HRV for early detection of neurocardiac risk in this population.

Methods

Artifact-free five-minute ECG recordings were analyzed using LabChart Version 8 (ADInstruments, Bella Vista, Australia) to derive HRV and ECG parameters. HRV assessment included both time-domain and frequency-domain measures, while ECG analysis comprised heart rate, PR interval, QRS duration, QT interval, and corrected QT interval (QTc). Demographic and clinical information were retrieved from departmental records for a total of 314 subjects. Following data quality assessment and exclusion of recordings with artifacts or incomplete data, HRV and ECG analyses were conducted on 278 subjects. Statistical analyses were performed using Jamovi macOS Pro (Version 2.6.45) and Statistics Kingdom, a freely accessible online statistical platform. Depending on the distribution and nature of the data, analyses included Spearman rank correlation, Welch’s t-test, and one-sample and two-sample t-tests.

Results

ECG findings demonstrated largely preserved cardiac conduction and repolarization parameters across the cohort, although inter-individual variability in repolarization amplitude was observed. In contrast, HRV analysis revealed subtle autonomic dysregulation, including among subjects presenting predominantly with autonomic symptoms, suggesting impaired parasympathetic modulation despite normal ECG findings. Several HRV indices differed significantly from normative reference values, indicating altered autonomic regulation.

Conclusions

HRV is a sensitive, noninvasive tool for detecting early autonomic dysfunction in neurologically symptomatic adults, irrespective of the underlying neurogenic presentation. While conventional ECG parameters remain largely preserved in cardiac-asymptomatic individuals, HRV can identify subclinical neurocardiac alterations that may not be evident on routine electrocardiographic assessment. These findings support the integration of HRV into neurological evaluation protocols to facilitate early neurocardiac risk stratification and preventive cardiovascular care.

## Introduction

Disorders of the nervous system encompass both the central nervous system (CNS) and peripheral nervous system (PNS), including their somatic and autonomic components, and often present with overlapping clinical features. While CNS disorders commonly manifest as headache, cognitive impairment, movement abnormalities, and gait disturbances, autonomic nervous system (ANS) involvement may result in cardiovascular, gastrointestinal, urogenital, and thermoregulatory dysfunction [1,2]. Emerging evidence highlights a close relationship between neurological and cardiovascular health, with both systems sharing common risk factors and pathophysiological mechanisms. Major neurological disorders, including stroke, epilepsy, Parkinson’s disease, multiple sclerosis, and Alzheimer’s disease, are frequently accompanied by cardiovascular comorbidities and varying degrees of autonomic dysfunction. These neurocardiac interactions may contribute to arrhythmias, impaired cardiac autonomic regulation, heart failure, and increased cardiovascular morbidity and mortality [3-5].

Headache and migraine are increasingly recognized as disorders involving complex interactions between central neural pathways and peripheral trigeminovascular and parasympathetic systems. More broadly, disturbances in sympathetic-parasympathetic balance are implicated in a range of neurological conditions and may predispose affected individuals to adverse cardiovascular outcomes. Acute neurological insults such as stroke can provoke neurohormonal activation, myocardial injury, and cardiac rhythm disturbances, whereas chronic neurological disorders and recurrent seizures are associated with persistent autonomic dysregulation and cardiovascular instability [2,4,6].

The neurocardiac connection is further illustrated by primary autonomic disorders, including pure autonomic failure, postural orthostatic tachycardia syndrome (POTS), and related dysautonomias. These conditions commonly present with orthostatic intolerance, syncope, gastrointestinal and urogenital dysfunction, and disturbances of thermoregulation, all of which may be associated with increased cardiovascular risk and reduced quality of life [7,8]. Patients with epilepsy often exhibit altered autonomic regulation, reflected by abnormalities in heart rate variability (HRV) measures. These changes are commonly characterized by a relative increase in sympathetic activity and impaired autonomic balance. Growing evidence suggests that such HRV abnormalities may serve as indicators of autonomic dysfunction and have been associated with an increased risk of sudden unexpected death in epilepsy (SUDEP) [9].

HRV has emerged as a reliable, noninvasive marker of ANS function, reflecting the dynamic balance between sympathetic and parasympathetic influences on the sinoatrial node. Reduced HRV has been linked to autonomic dysfunction and adverse cardiovascular outcomes across a variety of neurological and systemic disorders. Electrocardiography (ECG) provides complementary information regarding cardiac electrical activity, while HRV offers additional insight into subclinical autonomic disturbances that may precede overt cardiovascular disease [10,11]. For comparative interpretation, the widely cited normative HRV reference values reported by Nunan et al. are mostly used as the primary reference standard [12]. However, HRV parameters are influenced by demographic, ethnic, environmental, and methodological factors, and therefore regional or institution-specific reference values may provide additional clinical relevance.

Despite its potential clinical value, HRV assessment remains underutilized in routine neurological practice in India. Therefore, the present descriptive, data-driven study was undertaken to evaluate HRV and ECG parameters in neurologically symptomatic but cardiac-asymptomatic adults attending a tertiary care center in semi-urban Bihar, India. The objective was to identify early autonomic and electrophysiological markers of neurocardiac vulnerability before the development of clinically apparent cardiovascular disease.

## Materials and methods

Study design and ethical considerations

This data-driven retrospective study was approved by the Institutional Ethics Committee of AIIMS Patna and conducted in accordance with the ethical principles of the Declaration of Helsinki. The requirement for informed consent was waived because the study involved the analysis of anonymized retrospective data.

Study population

Demographic and clinical information and the ECG recording were retrieved from both digital and non-digital departmental records maintained by the Department of Physiology, AIIMS, Patna, an Institute of National Importance located in semi-urban Bihar, India. Records collected between April 2023 and April 2025 were reviewed for demographic characteristics (age and sex), clinical presentation, presenting neurological symptoms, relevant medical history, and medication use. A total of 314 participants fulfilled the eligibility criteria and were included in the demographic and clinical profile analysis. Of these, 278 participants had complete and artifact-free electrophysiological recordings and were included in the ECG and HRV analyses.

Electrophysiological data acquisition

Digital ECG recordings were obtained using a PowerLab 8/35 system (AD Instruments, Bella Vista, Australia) with a sampling frequency of 1024 Hz and analyzed using LabChart version 8 software. Five-minute artifact-free lead II ECG recordings were used to derive heart rate (HR), PR interval, QRS duration, QT interval, corrected QT interval (QTc), ST-segment height, and T-wave amplitude, providing measures of cardiac conduction, depolarization, and repolarization. HRV analysis was performed on five-minute artifact-free RR interval recordings in accordance with established Task Force recommendations [12]. Time-domain and frequency-domain HRV parameters, including SDNN, root mean square of successive RR interval differences (RMSSD), coefficient of variation of RR intervals (CVRR), percentage of adjacent RR intervals differing by more than 50 ms (pRR50), total power, low-frequency power (LF), high-frequency power (HF), normalized LF (LFnu), normalized HF (HFnu), and the LF/HF ratio, were calculated using LabChart software [12].

Statistical analysis

Data were compiled in Microsoft Excel and analyzed using Jamovi for macOS (version 2.6.45) [13] and a free online website for statistical analysis, Statistical Kingdom (Melbourne, Australia) [14]. Continuous variables were expressed as mean ± standard deviation (SD), while categorical variables were summarized as frequencies and percentages. The Shapiro-Wilk test was used to assess the normality of continuous variables. Welch’s t-test was employed for group comparisons to account for unequal sample sizes and variances. HRV parameters were compared with the internationally used reference and previously published institutional normative reference values [15]. Spearman’s rank correlation analysis was performed to evaluate associations between HRV and ECG parameters. Statistical significance was defined as a two-tailed p-value of < 0.05.

## Results

Study population

Of the 364 records screened, 314 contained complete demographic information, viz., age, gender, presenting neurological symptoms, and comorbidities; personal history was included in the study and is represented in Tables 1-2. The study cohort comprised 153 (48.7%) males and 161 (51.3%) females. Dizziness (79.0%), headache/migraine (56.7%), and hyperhidrosis (37.6%) were the most frequently reported symptoms, while hypertension (24.2%), obesity (13.1%), and diabetes mellitus (8.0%) were the most common comorbidities. The study population had a mean age of 44.0 ± 17.5 years (95% CI: 42.1-46.0 years), with ages ranging from nine to 87 years. The near-zero skewness (-0.033) indicates a relatively symmetric age distribution, while the negative kurtosis (-0.913) suggests a flatter distribution than a normal distribution. Although the Shapiro-Wilk test indicated a statistically significant deviation from normality (p < 0.001), this finding should be interpreted in the context of the large sample size, where minor departures from normality may achieve statistical significance.

**Table 1:** Demographic and clinical profile of the study participants (N = 314) Participants may report multiple symptoms, comorbidities, and personal history characteristics; therefore, percentages do not sum to 100%

Variable	N (%)
Gender	
Male	153 (48.7)
Female	161 (51.3)
Presenting symptoms	
Dizziness	248 (79.0)
Headache/migraine	178 (56.7)
Hyperhidrosis	118 (37.6)
Constipation	45 (14.3)
Bladder dysfunction	44 (14.0)
Syncope	34 (10.8)
Tingling sensation	22 (7.0)
Bronchospasm	18 (5.7)
Tremor	16 (5.1)
Chills	12 (3.8)
Weakness	11 (3.5)
Erectile dysfunction	4 (1.3)
Anxiety	4 (1.3)
Insomnia	3 (1.0)
Vertigo	3 (1.0)
Dryness of mouth	1 (0.3)
Dysarthria	1 (0.3)
Altered sensorium	1 (0.3)
Memory loss	1 (0.3)
Gait disturbances	1 (0.3)
Palpitation	1 (0.3)
Comorbidities	
Hypertension	76 (24.2)
Obesity	41 (13.1)
Diabetes mellitus	25 (8.0)
Cerebrovascular disease	7 (2.2)
Hypothyroidism	6 (1.9)
Personal history	
Alcohol use	2 (0.6)
Smoking/tobacco use	1 (0.3)

**Table 2:** Descriptive statistics of age distribution in the study population (N = 314) Data are presented as measures of central tendency and dispersion, including mean, median, standard deviation, range, and 95% CI for the mean. Distribution characteristics are described using skewness, kurtosis, and the Shapiro–Wilk test for normality CI: confidence interval

Descriptive statistics of age distribution	
N	314
Mean	44
Std. error mean	0.986
95% CI mean lower bound	42.1
95% CI mean upper bound	46
Median	46
Standard deviation	17.5
Minimum	9
Maximum	87
Skewness	-0.033
Std. error skewness	0.138
Kurtosis	-0.913
Std. error kurtosis	0.274
Shapiro-Wilk W	0.977
Shapiro-Wilk p	< 0.001

HRV findings

HRV analysis was performed in 278 participants (Table 3). The HRV measures demonstrated substantial inter-individual variability, with marked positive skewness and high kurtosis observed for most absolute time-domain and frequency-domain parameters, particularly total power, very-low-frequency power (VLF), LF, HF, standard deviation of RR intervals (SDRR), CVRR, and RMSSD. In contrast, normalized spectral indices (normalized LF and HF components (LF(nu) and HF(nu)) showed comparatively symmetric distributions. These findings indicate significant departures from normality and support the use of non-parametric statistical approaches and median-based interpretation when evaluating autonomic function in this cohort.

**Table 3:** Descriptive statistics of HRV parameters in the study cohort (n = 278) ᵃMore than one mode exists; only the first mode is reported The table summarizes the distribution of time-domain and frequency-domain HRV indices derived from five-minute resting ECG recordings. Data are presented as measures of central tendency (mean, median, mode), variability (standard deviation, variance, IQR, range), and distribution characteristics (skewness and kurtosis), together with 95% CI for the mean. Time-domain parameters include SDRR, CVRR, RMSSD, pRR50, and mean RR interval. Frequency-domain parameters include total power, VLF, LF, HF, normalized LF and HF components (LF(nu) and HF(nu)), and the LF/HF ratio Note: The 95% CI of the mean assumes that sample means follow a t-distribution with N − 1 degrees of freedom HRV: heart rate variability; IQR: interquartile range; CI: confidence interval; SDRR: standard deviation of RR intervals; CVRR: coefficient of variation of RR intervals; RMSSD: root mean square of successive RR interval differences; pRR50: percentage of adjacent RR intervals differing by more than 50 ms; VLF: very-low-frequency power; LF: low-frequency power; HF: high-frequency power; HF(nu) and LF(nu): normalized high- and low-frequency components; LF/HF: low-frequency to high-frequency ratio

Descriptive statistics of HRV parameters													
	SDRR	CVRR	RMSSD	PRR 50	Total power	VLF	LF	LF(nu)	HF	HF(nu)	LF/HF	RR interval	
N	278	278	278	278	278	278	278	278	278	278	278	278	
Missing	0	0	0	0	0	0	0	0	0	0	0	0	
Mean	45	0.0526	44.5	13.2	9207	1748	1813	44.3	5011	52.2	1.36	0.799	
Std. error mean	4.38	0.0041	5.82	1.22	4377	904	874	1.28	2419	1.19	0.113	0.00863	
95% CI mean lower bound	36.4	0.0446	33	10.9	591	-32.2	93	41.8	250	49.8	1.14	0.782	
95% CI mean upper bound	53.6	0.0607	55.9	15.6	17822	3529	3534	46.8	9772	54.5	1.58	0.816	
Median	32	0.0398	25	3.36	880	315	217	44.1	250	53.5	0.8	0.794	
Mode	3.31ᵃ	0.0230ᵃ	1.18ᵃ	0	11.1ᵃ	6.68ᵃ	2.18ᵃ	24.2	0.479ᵃ	65.6	0.369	0.518ᵃ	
Standard deviation	73.1	0.0684	97	20.3	72971	15079	14572	21.3	40328	19.9	1.89	0.144	
Variance	5340	0.0047	9403	411	5.32E+09	2.27E+08	2.12E+08	455	1.63E+09	396	3.58	0.0207	
IQR	23.7	0.0267	27.2	14.5	1646	567	404	32.1	522	28.3	1.12	0.183	
Range	742	0.7	908	99	1.05E+06	244201	182364	96.1	557524	89.1	18.5	0.842	
Minimum	3.31	0.007	1.18	0	11.1	6.68	2.18	2.07	0.479	5.31	0.0232	0.476	
Maximum	746	0.707	909	99	1.05E+06	244208	182366	98.2	557525	94.4	18.5	1.32	
Skewness	7.4	6.87	7.22	1.8	12	15.3	11.4	0.215	11.1	-0.214	4.71	0.535	
Std. error skewness	0.146	0.146	0.146	0.146	0.146	0.146	0.146	0.146	0.146	0.146	0.146	0.146	
Kurtosis	60.1	54.1	55.4	2.5	157	244	132	-0.665	137	-0.633	32	0.656	
Std. error kurtosis	0.291	0.291	0.291	0.291	0.291	0.291	0.291	0.291	0.291	0.291	0.291	0.291	

Time-domain HRV measures demonstrated substantial variability, with mean SDRR, RMSSD, and pRR50 values of 45.0 ms, 44.5 ms, and 13.2%, respectively. Frequency-domain measures also showed wide dispersion, with total power displaying marked variability across participants. Median LF(nu) and HF(nu) values were 44.1 and 53.5, respectively, with a median LF/HF ratio of 0.80. Absolute HRV parameters, particularly total power, VLF, LF, and HF, exhibited marked right-skewed distributions and high kurtosis. Shapiro-Wilk testing confirmed significant deviations from normality for all HRV parameters (p < 0.01). These findings indicate substantial inter-individual variability in autonomic function within the study cohort.

Table 4 presents a comparison of HRV parameters between the study cohort and internationally published normative reference values. HRV indices were compared with established international normative data to evaluate differences in autonomic modulation.

**Table 4:** Comparison with international reference normative HRV values Statistical comparison was performed using Welch’s t-test, and effect size was calculated to assess the magnitude of between-group differences. A p-value < 0.05 was considered statistically significant. Positive or negative t values indicate the direction of difference compared with the international reference population Interpretive note: The reduction in mRR with relative preservation of RMSSD suggests maintained short-term parasympathetic modulation despite altered overall autonomic variability. HRV parameters were interpreted considering known variations due to age, sex, ethnicity, recording methodology, and population characteristics HRV: heart rate variability; SD: standard deviation; mRR: mean RR interval; IQR: interquartile range; CI: confidence interval; SDRR: standard deviation of RR intervals; CVRR: coefficient of variation of RR intervals; RMSSD: root mean square of successive RR interval differences; pRR50: percentage of adjacent RR intervals differing by more than 50 ms; VLF: very-low-frequency power; LF: low-frequency power; HF: high-frequency power; LF(nu) and HF(nu): normalized low- and high-frequency components; LF/HF: low-frequency to high-frequency ratio

Parameter	Cohort, mean (SD)	Reference, mean (SD)	T statistics	Effect size	P-value
mRR (ms)	799 (86)	926 (90)	-24.4492	1.41	6.282e-72
SDRR	45 (53)	50 (16)	-4.8874	0.3	5.144e-7
RMSSD	44.5 (5.82)	42 (15)	2.7761	0.17	0.005506
LF	1,813 (874)	519 (291)	2157.2518	130.22	0
LF(nu)	44.3 (1.28)	52 (10)	-12.8368	0.77	1.397e-37
HF	5,011 (2419)	657 (777)	88.0759	5.32	0
HF(nu)	52.2 (1.19)	40 (10)	20.3391	1.23	0
LF/HF	1.36(0.11)	2.8 (26)	-0.9234	0.056	0.3558

When compared with previously published international normative reference values, the study cohort demonstrated a significantly higher resting heart rate and lower mean RR interval and SDRR, indicating reduced overall heart rate variability. In contrast, RMSSD values were modestly but significantly higher than reference values. Frequency-domain analysis showed significantly higher absolute LF and HF power values. Normalized spectral components demonstrated lower LF(nu) and higher HF(nu) values compared with normative data, whereas the LF/HF ratio did not differ significantly. Overall, the findings suggest altered autonomic regulation characterized by reduced global HRV with relative preservation of parasympathetic modulation.

Table 5 presents a comparison of HRV parameters between the study cohort and institutional healthy reference subjects.

**Table 5:** Comparison of HRV parameters between the study cohort and institutional healthy reference subjects Institutional reference values were derived by averaging the sex-specific normative values reported separately for males and females in the reference article. Comparisons were restricted to parameters for which reference data were available. Effect size is reported as Cohen’s d HRV: heart rate variability; SD: standard deviation; RMSSD: root mean square of successive RR interval differences; LF: low-frequency power; HF: high-frequency power; LF/HF: low-frequency to high-frequency ratio

Parameter	Cohort (n = 278), mean (SD)	Institute reference (n = 278), mean (SD)	T statistics	Effect size	P-value
mRR (ms)	799 (86)	763.125 (126.695)	2.7371	0.36	0.006939
RMSSD	44.5 (5.82)	45.305 (24.205)	-0.2464	0.058	0.7297
LF	1,813 (874)	626.96 (539.35)	16.1873	1.49	0
HF	5,011 (2419)	1046.96 (1035.425)	22.6213	1.87	0
LF/HF	1.36 (0.11)	0.87 (1.49)	3.461	0.61	0.0007661

Descriptive statistics of resting electrocardiographic (ECG) parameters of the study cohort

A total of 278 participants had complete and artifact-free ECG recordings available for analysis. The mean RR interval was 0.799 ± 0.144 seconds, corresponding to a mean heart rate of 78.0 ± 14.3 beats/minute. Mean PR interval, QRS duration, QT interval, and QTc were 0.155 seconds, 0.076 seconds, 0.325 seconds, and 0.367 seconds, respectively, indicating generally preserved cardiac conduction and ventricular repolarization.

ST-segment height and T-wave amplitude demonstrated considerable variability, with a small number of extreme values. Shapiro-Wilk testing showed significant deviation from normality for all ECG parameters (p < 0.01), with the greatest departures observed for ST-segment and T-wave amplitudes (p < 0.001). Descriptive statistics for all ECG variables are summarized in Table 6.

**Table 6:** Descriptive statistics of resting ECG parameters of the study cohort ^ᵃ^More than one mode existed; only the first mode is reported Data are presented as measures of central tendency, dispersion, and distribution for resting ECG parameters derived from five-minute artifact-free recordings. Variables include average HR, PR interval, QRS duration, QT interval, QTc, ST-segment height, and T-wave amplitude. Marked skewness and kurtosis observed for ST-segment height, T-wave amplitude, and QRS duration indicate non-normal distributions and substantial inter-individual variability. These findings support the use of non-parametric statistical approaches for subsequent analyses. Values are reported as mean, median, standard deviation, IQR, range, and 95% CI of the mean. The 95% CI assumes a t-distribution with N − 1 degrees of freedom ECG: electrocardiography; HR: heart rate; QTc: corrected QT interval; IQR: interquartile range; CI: confidence interval

Descriptive statistics of resting electrocardiographic (ECG) parameters							
	Avg HR	PR interval (s)	QRS interval (s)	QT interval (s)	QTc (s)	ST height (mV)	T amplitude (mV)
N	278	278	278	278	278	278	278
Missing	0	0	0	0	0	0	0
Mean	78	0.155	0.0761	0.325	0.367	0.205	0.453
Std. error mean	0.855	0.00141	0.00116	0.0024	0.00254	0.155	0.218
95% CI mean lower bound	76.3	0.153	0.0738	0.32	0.362	-0.101	0.025
95% CI mean upper bound	79.6	0.158	0.0784	0.33	0.372	0.511	0.881
Median	75.8	0.155	0.0731	0.326	0.37	0.0151	0.228
Mode	68.6ᵃ	0.128ᵃ	0.0634ᵃ	0.305ᵃ	0.373	-0.206ᵃ	0.168ᵃ
Standard deviation	14.3	0.0235	0.0194	0.0392	0.0423	2.59	3.63
Variance	203	5.51E-04	3.74E-04	0.0015	0.00179	6.72	13.2
IQR	17.6	0.0335	0.0131	0.0383	0.0335	0.0658	0.17
Range	79.2	0.119	0.167	0.278	0.443	42	60.9
Minimum	46.8	0.109	0.0507	0.142	0.142	-0.206	-0.383
Maximum	126	0.228	0.218	0.421	0.585	41.8	60.6
Skewness	0.653	0.367	4.22	-1.16	-1.08	15.3	16.6
Std. error skewness	0.146	0.146	0.146	0.146	0.146	0.146	0.146
Kurtosis	0.5	-0.216	25.6	4.39	8.6	242	275

ECG comparison with reference

Table 7 presents the results of one-sample Wilcoxon signed-rank tests for ECG parameters derived from five-minute artifact-free resting recordings.

**Table 7:** One-sample Wilcoxon signed-rank analysis of ECG parameters obtained from five-minute resting ECG recordings (n = 278) Data are presented as the Wilcoxon W statistic, two-sided p-value, estimated median (reported as mean difference by the software), 95% CI, and rank-biserial correlation effect size. Significant p-values indicate that the observed parameter values differ from the hypothesized median value. ECG variables evaluated included RR interval, average HR, PR interval, QRS duration, QT interval, corrected QT interval (QTc), ST-segment height, and T-wave amplitude. Effect size was estimated using the rank-biserial correlation. Hₐ: μ ≠ 0 (two-tailed alternative hypothesis) Note: Since all variables were tested against a null value of 0, statistical significance is expected for physiological ECG parameters. Therefore, the descriptive statistics and clinical interpretation of ECG indices are of greater relevance than the significance of the one-sample test itself ECG: electrocardiography; HR: heart rate; QTc: corrected QT interval; CI: confidence interval

One-Sample Wilcoxon Signed-Rank Analysis								
					95% CI			
		Statistic	p	Mean difference	Lower	Upper		Effect size
RR interval	Wilcoxon W	38781	< 0.001	0.7939	0.77731	0.8109	Rank-biserial correlation	1
Avg HR	Wilcoxon W	38781	< 0.001	77.07	75.40996	78.7351	Rank-biserial correlation	1
PR interval (s)	Wilcoxon W	38781	< 0.001	0.1546	0.1517	0.1575	Rank-biserial correlation	1
QRS interval (s)	Wilcoxon W	38781	< 0.001	0.0735	0.07224	0.0748	Rank-biserial correlation	1
QT interval (s)	Wilcoxon W	38781	< 0.001	0.3265	0.32283	0.3303	Rank-biserial correlation	1
QTc (s)	Wilcoxon W	38781	< 0.001	0.37	0.36667	0.3733	Rank-biserial correlation	1
ST height (mV)	Wilcoxon W	25799	< 0.001	0.015	0.00907	0.0212	Rank-biserial correlation	0.33
T amplitude (mV)	Wilcoxon W	37385	< 0.001	0.2296	0.214	0.2456	Rank-biserial correlation	0.928

A one-sample Wilcoxon signed-rank test was performed to examine whether ECG parameters differed significantly from the reference value (Hₐ: μ ≠ 0). The analysis demonstrated highly significant deviations across all ECG variables (all p < 0.001) as depicted in Table 6. The RR interval showed a mean difference of 0.794 s (95% CI: 0.777-0.811) with a very large effect size (rank-biserial correlation = 1.000), indicating a consistent shift across the cohort. Similarly, average heart rate differed significantly from the reference (mean difference: 77.07 bpm; 95% CI: 75.41-78.74), again with a maximal effect size.

All major conduction and repolarization indices-including PR interval, QRS duration, QT interval, and QTc-demonstrated statistically significant prolongation, each with narrow confidence intervals and large to maximal effect sizes (rank-biserial correlation = 1.000), reflecting a uniform alteration in cardiac electrical activity within the study population. ST segment height also differed significantly (mean difference: 0.015 mV; 95% CI: 0.009-0.021), though with a small-to-moderate effect size (rank-biserial correlation = 0.330), suggesting greater inter-individual variability. In contrast, T-wave amplitude showed a significant difference with a large effect size (mean difference: 0.230 mV; 95% CI: 0.214-0.246; rank-biserial correlation = 0.928). Overall, these findings indicate robust and clinically meaningful deviations in ECG parameters in the cohort, supporting the presence of altered cardiac electrophysiological profiles.

Correlation matrix

HRV and ECG

Spearman correlation matrix findings between HRV and ECG parameters in the cohort demonstrated strong and statistically significant positive correlations among time-domain HRV indices. SDRR showed very high associations with CVRR (ρ = 0.947, p < 0.001), RMSSD (ρ = 0.854, p < 0.001), PRRX (ρ = 0.830, p < 0.001), and total power (ρ = 0.937, p < 0.001). Similarly, RMSSD demonstrated a very strong correlation with PRRX (ρ = 0.946, p < 0.001), indicating internal consistency among parasympathetic markers of HRV. In the frequency domain, total power correlated strongly with VLF (ρ = 0.860), LF (ρ = 0.911), and HF (ρ = 0.879), all p < 0.001. LF and HF also showed a strong positive association (ρ = 0.759, p < 0.001). Normalized LF (LF(nu)) showed significant negative correlations with RMSSD (ρ = −0.458, p < 0.001) and HF (ρ = −0.455, p < 0.001), while HF(nu) correlated strongly and negatively with LF(nu) (ρ = −0.900, p < 0.001), reflecting sympatho-vagal balance dynamics.

The LF/HF ratio demonstrated strong inverse relationships with RMSSD (ρ = −0.448) and HF (ρ = −0.463), and a near-perfect positive correlation with LF(nu) (ρ = 0.992), all p < 0.001. As expected, RR interval and average heart rate were almost perfectly inversely correlated (ρ = −0.998, p < 0.001). However, RR interval showed no significant correlation with HRV spectral parameters, indicating relative independence in this.

Regarding ECG parameters, PR interval showed a weak but significant positive correlation with RR interval (ρ = 0.169, p = 0.005) and a corresponding negative correlation with average heart rate (ρ = −0.169, p = 0.005). QT interval correlated strongly and positively with RR interval (ρ = 0.577, p < 0.001) and negatively with heart rate (ρ = −0.581, p < 0.001). QTc showed moderate positive correlations with QT interval (ρ = 0.451, p < 0.001) and heart rate (ρ = 0.351, p < 0.001), and a negative correlation with RR interval (ρ = −0.356, p < 0.001).ST segment height demonstrated modest but significant negative correlations with QT interval (ρ = −0.186, p = 0.002) and QTc (ρ = −0.294, p < 0.001). No significant correlations were observed between T-wave amplitude and HRV or ECG parameters.

Overall, the matrix highlighted robust internal correlations within HRV domains, clear physiological relationships between heart rate, RR interval, and repolarization indices, and limited direct associations between HRV measures and surface ECG intervals, supporting the role of HRV as a sensitive marker of autonomic modulation preceding overt ECG changes.

Gender-wise comparison of ECG and HRV parameters in the study cohort

Comparative analysis between the two genders revealed statistically significant gender-based differences across most ECG and HRV parameters.

ECG Parameters

Males demonstrated a significantly higher resting heart rate, longer PR interval, and higher QTc values compared to females. However, the QTc values were significantly higher in males (p < 0.001), with a large effect size (d = 1.47). The difference in QRS and QT intervals was not statistically significant.

HRV Parameters

All major time-domain HRV indices - SDRR, CVRR, and RMSSD - were significantly higher in males than females (all p < 0.001), each demonstrating large effect sizes (d range: 2.49-3.08). However, pRR50 did not differ significantly between genders (p = 0.149), indicating comparable short-term vagal modulation. Frequency-Domain HRV indices (Total power, VLF, LF, LF(nu), HF power and LF/HF ratio) were all markedly higher in males (all p < 0.001), except HF(nu), which was significantly higher in females (p < 0.001), reflecting gender-specific sympathovagal balance. With large effect sizes (d = 1.88-2.45), these findings suggest greater overall autonomic variability in males, as shown in Table 8.

**Table 8:** Gender-wise comparison of ECG and HRV parameters in the study cohort Between-group comparisons were performed using the independent samples t-test. Effect size is reported as Cohen’s d and interpreted according to conventional thresholds (small: 0.2–0.49, moderate: 0.5–0.79, large: ≥0.8). A p value < 0.05 was considered statistically significant ECG: electrocardiography; HRV: heart rate variability; SD: standard deviation; HR: heart rate; PR interval: atrioventricular conduction time; QRS duration: ventricular depolarization duration; QT interval: ventricular depolarization–repolarization duration; QTc: heart rate-corrected QT interval; SDRR: standard deviation of RR intervals; CVRR: coefficient of variation of RR intervals; RMSSD: root mean square of successive differences between adjacent RR intervals; pRR50: percentage of adjacent RR intervals differing by more than 50 ms; TP: total power; VLF: very low-frequency power; LF: low-frequency power; HF: high-frequency power; LF (nu) and HF (nu): normalized units of low- and high-frequency power; LF/HF: ratio of low-frequency to high-frequency power

Parameter	Mean (SD) (M = 149)	Mean (SD) (F = 129)	T test	Effect size	P-value
HR (bpm)	79.7 (1.23)	75.7 (1.14)	27.9708	Large, 3.36	0
PR interval (s)	0.151 (0.00241)	0.0755 (0.00108)	328.3549	39.49	0
QRS duration (s)	0.0767 (0.00196)	0.0755 (0.00108)	6.187	0.74	2.205e-9
QT interval (s)	0.320 (0.00316)	0.329 (0.00426)	-20.1664	2.43	3.28e-56
QTC (s)	0.366 (0.00385)	0.00311 (0.362)	12.2392	1.47	0
SDRR	53.5 (7.94)	35.1 (1.98)	25.6337	3.08	0
CVRR	0.0595 (0.00738)	0.0447 (0.00222)	21.9297	2.64	0
RMSSD	53.7 (10.6)	33.9 (2.62)	20.6713	2.49	0
PRR50	13.1 (1.71)	13.4 (1.73)	-1.4509	0.17	0.149
TP	15,801 (8,137)	1,591 (238)	19.8225	2.38	0
VLF	2,824 (1,684)	506 (68.7)	15.6188	1.88	0
LF	3,076 (1,625)	355 (62.2)	19.0016	2.29	0
LF(nu)	46.5 (1.77)	41.8 (1.83)	21.7348	2.61	0
HF	8,757 (4,496)	685 (133)	20.3789	2.45	0
HF(nu)	49.8 (1.62)	54.9 (1.74)	-25.2915	3.04	7.662e-74
LF/HF	1.39 (0.117)	1.32 (0.204)	3.4383	0.43	0.0007139

## Discussion

This data-driven observational study evaluated autonomic and electrophysiological cardiac alterations in neurologically symptomatic but cardiac-asymptomatic adults using short-term HRV and resting ECG analysis. The principal finding was that HRV demonstrated significant alterations in autonomic regulation despite largely preserved conventional ECG parameters, highlighting the potential role of HRV in identifying early neurocardiac vulnerability.

HRV findings and autonomic regulation

Time-Domain Measures and Autonomic Balance

Time-domain HRV parameters demonstrated considerable variability, particularly SDRR, with a mean value of approximately 45 ms and a median value of approximately 32 ms. Although average values were not markedly abnormal, the wide dispersion and right-skewed distribution indicate the presence of a subgroup with reduced autonomic adaptability. SDNN is considered an important marker of overall HRV and is widely used for cardiovascular risk assessment, particularly when derived from long-duration recordings [16]. The observed variability in HRV parameters, including reduced global variability in a subset of participants, may reflect altered autonomic regulation associated with neurological symptoms. Changes in parasympathetic and sympathetic modulation have been described in neurological conditions such as migraine, stroke, movement disorders, and autonomic dysfunction syndromes [17,18]. However, interpretation of short-term HRV measures should consider recording duration and population-specific reference values.

Frequency-Domain Measures and Autonomic Balance

Frequency-domain analysis demonstrated marked inter-individual variability, particularly in total power, LF, and HF components. The distribution pattern of these parameters suggests substantial heterogeneity in autonomic function within the study population. Normalized frequency-domain measures showed reciprocal changes between LF(nu) and HF(nu), indicating altered sympathovagal modulation rather than isolated changes in absolute spectral power. These findings emphasize the importance of evaluating the distribution and variability of HRV parameters rather than relying only on mean values, especially in heterogeneous neurological populations. HRV interpretation using both absolute and normalized measures may provide a more comprehensive assessment of autonomic function [19].

ECG Findings and Neurocardiac Assessment

Conventional ECG parameters, including PR interval, QRS duration, QT interval, and QTc, were largely within expected ranges at the cohort level, suggesting preserved cardiac conduction and repolarization in most participants. However, variability in repolarization-related parameters, including QTc, ST-segment height, and T-wave amplitude, suggests that subtle electrophysiological alterations may exist in a subset of individuals. The relative preservation of resting ECG findings despite altered HRV parameters supports the concept that autonomic dysfunction may precede detectable abnormalities on routine ECG. Thus, HRV may provide additional information regarding early neurocardiac changes that may not be apparent through conventional cardiac assessment alone [20].

Association Between HRV and ECG Parameters

The observed strong correlations among time-domain and frequency-domain HRV measures demonstrate internal consistency of autonomic indices. HRV parameters showed limited correlation with routine ECG intervals, whereas QT and QTc demonstrated significant association with heart rate, suggesting that autonomic alterations may occur independently of overt electrical abnormalities.

The inverse relationship between LF/HF ratio and HF(nu) further supports the physiological relationship between sympathetic and parasympathetic components. Similar associations between HRV parameters have been reported previously, including correlations between SDNN and VLF power, pNN50/RMSSD and HF power, and RMSSD with parasympathetic indices [21-23]. Previous studies have also evaluated associations between ECG parameters and HRV measures; however, differences in statistical approaches, such as the use of Pearson correlation in normally distributed datasets, may influence observed relationships. In the present study, Spearman’s correlation was selected because several variables demonstrated non-normal distributions, highlighting the importance of selecting statistical methods according to data characteristics.

Sex-Related Considerations

Sex-related differences in HRV have been reported in previous studies, with variations in autonomic balance and HRV parameters observed between males and females [24]. Similar differences in autonomic measures were also observed in our cohort. These findings may reflect underlying physiological differences between sexes and may also contribute to variations in susceptibility to stress-related neurological and cardiovascular changes. Further studies with sex-specific analysis and longitudinal follow-up are needed to better understand the clinical significance of these differences.

Limitations 

The retrospective design of the study limits causal inference and control over potential confounders. Comparisons were performed using published international and institutional reference values rather than concurrently recruited healthy controls. The institutional reference values were derived from averaged sex-specific normative data reported in the reference article, and not all HRV parameters could be compared due to limited availability of reference data. Additionally, information on factors that may influence HRV and ECG measures, such as medications, lifestyle factors, and disease severity, was not uniformly available.

## Conclusions

In neurologically symptomatic but cardiac-asymptomatic adults, HRV analysis reveals significant autonomic dysfunction even when conventional ECG findings remain largely within normal limits. The marked dispersion and deviation of HRV parameters from established normative values suggest the presence of subclinical alterations in autonomic regulation, highlighting an underlying neurocardiac vulnerability that may not be detectable through routine electrocardiographic assessment alone. These findings underscore the complementary value of HRV in evaluating neurocardiac interactions and identifying individuals at potential risk of future cardiovascular complications. When used alongside standard ECG assessment, HRV serves as a powerful, noninvasive tool for the early detection of autonomic dysregulation and cardiac risk. Incorporating HRV into the routine evaluation of patients with neurological symptoms may facilitate earlier recognition of neurocardiac involvement and support more comprehensive clinical assessment and risk stratification. Future prospective studies incorporating age- and sex-matched healthy controls are needed to validate these findings and establish population-specific normative values. Longitudinal investigations may help determine the clinical significance of ECG-HRV alterations and their relationship with neurological symptom burden, disease progression, and autonomic dysfunction. Integration of multimodal autonomic assessments and advanced analytical approaches may further improve the identification of subclinical cardiac autonomic abnormalities in neurologically symptomatic individuals.
